# Survey of *Anaplasma phagocytophilum* and *Anaplasma* sp. ‘Omatjenne’ infection in cattle in Africa with special reference to Ethiopia

**DOI:** 10.1186/s13071-018-2633-y

**Published:** 2018-03-09

**Authors:** Sori Teshale, Dirk Geysen, Gobena Ameni, Pierre Dorny, Dirk Berkvens

**Affiliations:** 1 Addis Ababa University, College of Veterinary Medicine and Agriculture, Bishoftu, Ethiopia; 2 Department of Biomedical Sciences, Institute of Tropical Medicine, Antwerp, Belgium; 3 Ghent University, Faculty of Veterinary Medicine, Merelbeke, Belgium; 4 Addis Ababa University, Akililu Lemma Institute of Pathobiology, Addis Ababa, Ethiopia; 5 Ghent University, Faculty of Bio-engineering Sciences, Ghent, Belgium

**Keywords:** *Anaplasma phagocytophilum*, *Anaplasma* sp., ‘Omatjenne’, Cattle, Africa, Ethiopia

## Abstract

**Background:**

As evidence of the infection of domestic animals by *Anaplasma phagocytophilum* and *Anaplasma* sp. ‘Omatjenne’ is presently becoming available, understanding the epidemiological and ecological significance of infection is important to quantify the clinical and socio-economic impact of the diseases they cause.

**Methods:**

The first aim of this study was to analyse the occurrence of *A. phagocytophilum* and *Anaplasma* sp. ‘Omatjenne’ in cattle samples collected from selected African countries using a polymerase chain reaction and restriction enzyme fragment length polymorphism. Secondly, this study was aimed at the molecular identification of *Ehrlichia* spp. and *Anaplasma* spp. infection in ruminants raised under different production systems in selected sites in central Ethiopia.

**Results:**

In total, 695 samples from cattle in six African countries were analysed. Overall, 45 positive results were obtained for *Anaplasma* sp. ‘Omatjenne’ (6.47%) and 19 for *A. phagocytophilum* (2.73%). *Anaplasma* sp. ‘Omatjenne’ was detected in all countries except Tanzania while *A. phagocytophilum* was detected only in samples from Ethiopia. The proportion of samples tested positive for *Anaplasma* sp. ‘Omatjenne’ ranged from 1.2% in Morocco to 16% in Rwanda. The occurrence of both agents is now confirmed in African cattle. For the survey in Ethiopia a semi-nested *16S* rDNA polymerase chain reaction followed by restriction fragment length polymorphism was used for the identification of *Ehrlichia* spp. and *Anaplasma* spp. in blood samples. Randomly selected samples were also analysed by *pCS20* polymerase chain reaction for the detection of *E. ruminantium*. Positive results were obtained for *E. ruminantium* and five species of *Anaplasma* including a zoonotic species*.* To our knowledge, this is the first report of infection of domestic ruminants with *A. phagocytophilum*, *A. ovis* and *Anaplasma* sp. ‘Omatjenne’ in Ethiopia.

**Conclusion:**

The present study showed widespread occurrence of *Anaplasma* sp. 'Omatijenne' in African cattle and five *Anaplasma* species in Ethiopia.

## Background

*Anaplasma phagocytophilum* is a recently emended species of bacteria that comprises *Ehrlichia phagocytophila*, *Ehrlichia equi* and the agent of human granulocytic ehrlichiosis [1]. The bacterium appears to be a generalist, infecting a wide range of domestic and wild animals (causing tick-borne fever) and humans (causing human granulocytic anaplasmosis) [2]. Most outbreaks of tick-borne fever have been recorded in sheep flocks and cattle herds but isolated outbreaks have also been documented in goats. Although mortality and morbidity due to *A. phagocytophilum* infection is low in animals, economic losses due to a reduction in milk yield, abortion, infertility and reduced weight gains have been observed in pastured animals [3, 4]. Death can also occur in weaker animals if left untreated [5]. Since its recognition, an escalating number of human cases of *A. phagocytophilum* have been reported in the USA, Europe, Middle East and Asia, causing hospitalisation of 36% in the USA and a mortality of 26.5% in China [3]. Although tick attachment is thought to be the main route of human infection, contact with infected animal blood and prenatal infection has been reported [6]. Despite its veterinary and public health importance, data on the occurrence of *A. phagocytophilum* are lacking for the African continent. Only a few published articles reported its occurrence and detection of the bacterium was coincident with detection of other tick-borne pathogens. For instance, Muhanguzi et al. [7] detected DNA of this pathogen in 2.7% of cattle blood in Uganda while Sarih et al. [8] detected its DNA in two *Ixodes ricinus* specimens in Tunisia. Recently, the bacterium was detected in ticks in Ethiopia [9]. Apart from these, no systematic and pre-designed epidemiological studies were undertaken to explore *A. phagocytophilum*. Empirically, several tick associated febrile diseases, whose etiologic agents remain obscure, have been reported frequently by field veterinarians. Elsewhere, it has been shown that 8.6% of such tick-associated febrile diseases (15% in horses and 6% in dogs) are caused by *A. phagocytophilum* [10]. The possibility of *A. phagocytophilum* being involved in such cases in Africa cannot be ruled out. Investigation into the occurrence of *A. phagocytophilum* from different areas of Africa is needed.

*Anaplasma* sp. ‘Omatjenne’, a rickettsia belonging to the family *Anaplasmataceae*, was described for the first time by Allsopp et al. [11] and was detected in healthy Boer goats in heartwater-free areas of South Africa using molecular methods. The distribution and epidemiology of this genotype of rickettsia has not been investigated in Africa. However, infection with it has been reported in wild and domestic animals in Africa and the Mediterranean. It was detected in 1.9% of cattle in Uganda [7] and in Turkey [12]. It has also been reported in the Nyala (*Tragelaphus angasii*) in four game ranches sharing grazing areas with cattle in KwaZulu-Natal [13] and African buffaloes (*Syncerus caffer*) in the Kruger National Park and in the Hluhluwe-iMfolozi Park [14]. It was also detected in *Amblyomma* ticks collected from animals in Ethiopia [9]. Since the role of this *Ricketssia* in producing clinical disease remains obscure, study on its occurrence in Africa is required.

The epidemiological and ecological significance of infection with *A. phagocytophilum* has not been elucidated and the importance of *Anaplasma* sp. ‘Omatjenne’ in causing disease has not been clarified despite its occurrence in ruminants. The first aim of this study was to analyse the occurrence of *A. phagocytophilum* and *Anaplasma* sp. ‘Omatjenne’ in bovine samples collected from several African countries.

Secondly, a detailed field survey was conducted trying to elucidate the occurrence of infection by species of *Erhlichia* and *Anaplasma* in cattle, sheep and goats in central Oromia (Ethiopia) using molecular methods. The information was used to answer the following questions: (i) Which *Ehrlichia* spp. and *Anaplasma* spp. infect ruminants in central Ethiopia? and (ii) What is the prevalence of infection of these pathogens in ruminants in central Ethiopia? Ticks are widespread in all agro-ecological zones of Ethiopia [15–17] and empirical evidence shows heartwater and anaplasmosis problems in areas where *Amblyomma* spp. and *Rhipicephalus* spp. are encountered. However, epidemiological investigation of tick-borne diseases is either absent or inadequate in the country [18]. The available information is obsolete because the results are based on serology and microscopy with obvious limitations [19]. Only limited molecular studies have been carried out so far by Tomassone et al. [20], Kumsa et al. [21] and Teshale et al. [9], mostly in ticks. Since Ethiopia has diverse agro-climatic regions that support several vector species, mixed infections with *Ehrlichia* spp. and *Anaplasma* spp. are common. Molecular characterisation is essential for accurate differential diagnosis and detection of mixed infections of species belonging to the two genera. This leads to a better understanding of the epidemiology in order to minimise losses incurred in livestock during genetic improvement and translocation programs. Making an inventory of tick-borne *Erhlichia* spp. and *Anaplasma* spp. is an important pre-requisite to understand their role in constraining ruminant production.

## Methods

### International study

Samples used in this study were DNA samples extracted from bovine blood collected from selected African countries. The samples (238 in total), originating from Ivory Coast (*n* = 53), Morocco (*n* = 81), Rwanda (*n* = 50), Tanzania (*n* = 17) and Zambia (*n* = 37), were obtained from the Department of Biomedical Science, Institute of Tropical Medicine (Antwerp, Belgium) and were analysed using *16S* rDNA PCR-RFLP as described by Teshale et al. [9].

### Oromia survey

#### Study areas

The study was carried out in five livestock premises: Bishoftu, Bako, Habernosa, Alage and Adami-Tullu. They are distributed in three districts (Ada’a-Liban, Bako-Tibbe and Adami-Tullu-Jiddo-Kombolcha) in central Oromia (Ethiopia) (Table 1). Selection of the study sites was based on previous clinical and post-mortem reports of heartwater using microscopic examination of brain squash smears at the National Animal Health Research and Diagnostic Centre (Sabata, Ethiopia) [22], serological reports of anaplasmosis at the International Livestock Research Institute (Addis Ababa, Ethiopia) [18] and molecular evidence of infection in ticks in the area [9, 21].

**Table 1:** Description of the study sites in the three districts in Ethiopia

Description	Ada’a Liban	Bako	Adami-Tullu-Jiddo-Kombolcha
Location	9°N, 4°E	9°8’ N, 37°5′E	7°9’N, 38°7′E
Mean temperature (°C)	8.5–30.7	13.5–27.9	22.3–28.8
Mean annual RF (mm)	1156	1227	760.9
Mean relative humidity (%)	61.3	85	63
Mean altitude (m above sea level)	1550	1650	1650
Farming type	Mixed	Mixed	Livestock based
Main livestock raised	Cattle, sheep, goats	Cattle and sheep	Cattle and goats
Premises selected	Bishoftu farms	Bako Research farm	Habernosa ranch, Alage dairy farm, Adami Tullu farm
Animals sampled	Cattle (*n* = 125), sheep (*n* = 125), goats (*n* = 51)	Cattle (*n* = 149), sheep (*n* = 164)	Cattle (*n* = 183), goats (*n* = 125)
Samples collected	Blood and ticks	Blood and ticks	Blood and ticks

Heartwater was suspected to occur in exotic dairy cattle in Ada’a-Liban District, in and around Bishoftu on several farms. Bishoftu is one of the international tourist destinations, located in the Eastern Shewa Zone of the Oromia Regional State southeast of Addis Ababa. It is the main town of Ada’a-Liban district, situated on two international trade routes: (i) the Franco-Ethio-Djibouti railway and (ii) the Addis-Moyale-Nairobi international asphalted route. It is one of the main milk producing areas for Addis Ababa and is known for its commercial livestock production. Commercial dairy farms raising exotic dairy cattle (Holstein cattle and their crosses with zebu) and smallholder farmers rearing zebu cattle as well as Menz sheep and local goats were included in the study. Data were collected in February 2012.

The Bako Agricultural Research Centre with a history of clinical heartwater cases in lambs and calves, detected by pathology, was also selected for this study. Teshale et al. [9] detected DNA of *E. ruminantium* and *Anaplsma* spp. in ticks collected from cattle raised on this premise. The Bako region has a warm and humid climate that supports survival and proliferation of several tick species. The main vegetation of the area is a forest type, which is favorable for wildlife. Mixed crop-livestock production is the economic mainstay for communities in the area. Data collection was carried out in December 2012.

The remaining three selected livestock premises (Habernosa, Adami-Tullu and Alage farms) are found in Adami-Tullu-Jiddo-Kombolcha district. The district is located in the middle of the Rift Valley of Ethiopia to the south of Addis Ababa. Data from this district were collected in January 2012. The main climate type of the district is semi-arid and livestock production is the dominant farming system. Dairy cattle are mostly reared in small-scale dairy operations under different levels of management. These three livestock premises were selected based on their previous history of mortality of dairy cattle due to tick-borne diseases [22]. Habernosa is a private ranch where exotic (Holstein) animals and their crosses with Borana cattle are raised and distributed to nearby smallholder farmers. On this ranch heartwater was confirmed in 17 of 40 cattle that died after showing clinical signs of the disease [22]. The occurrence of *Ehrlichia* spp. and *Anaplasma* spp. in ticks collected from this ranch was confirmed by molecular techniques [9]. Currently the ranch has introduced goats of the Arsi breed obtained from the south-eastern highlands. The goats are at risk of tick-borne infections since they are introduced from areas where tick challenge is low. Adami-Tullu is an agricultural research centre breeding mainly Arsi goats and cattle for distribution to nearby farmers. Alage is a small village with commercial dairy and swine farms. Samples were collected from dairy cattle as clinical cases of heartwater have been experienced on this farm.

#### Sample collection

For the survey in Ethiopia, blood samples were collected from cattle, sheep and goats using EDTA coated vacutainer tubes. In addition, information on the type of farms (commercial, ranch or smallholder) and the type and frequency of tick control was recorded. Convenience sampling was used throughout with the primary aim to detect and describe the occurrence tick-borne diseases. Statistical analysis was not envisaged as controlling for possible confounders proved impossible. Overall, 922 blood samples (457 cattle, 289 sheep and 176 goats) were collected. Ticks infesting the study animals were also collected and identified based on morphological identification keys described in [23]. The identification was carried out in the Veterinary Parasitology Laboratory of the College of Veterinary Medicine and Agriculture of the Addis Ababa University (Bishoftu, Ethiopia). DNA samples were extracted from whole blood using Gentra (Puregene® 2010, Qiagen, Mainz, Germany) as described by the manufacturer and stored at -18 °C before transferring them to the Biomedical Sciences Department of the Institute of Tropical Medicine (Antwerp, Belgium) for molecular characterisation.

### Molecular analysis of the samples

#### 16S rDNA polymerase chain reaction

A semi-nested PCR described by [9] was used to amplify a fragment of *16S* rRNA gene. Primers designated EHR 16SD [24] and EBR3 [9] were used for the first round of PCR amplification. For the second round of amplification the same EHR 16SD was used as forward external primer while EBR2 [9] was used as internal primer. Details of the primers used are presented in Table 2. The reaction mix consisted of HotStartTaq Master Mix (2.5 units of DNA polymerase, PCR buffer containing 1.5 mM MgCl_2_ and 200 μM of each dNTP), 0.2 μM of each primer and PCR water. The PCR reaction was carried out in a total volume of 25 μl using a programmable thermocycler (T3 thermal cycler Biometra® Kit, Westburg, the Netherlands). All PCR products were visualised by gel electrophoresis in TAE buffer (0.04 M Tris, 0.4 mM EDTA, pH = 7.7–8.8) using 2% agarose at 100 V for 20 min and staining with ethidium bromide. Throughout the PCR procedures, a PCR mix without DNA template was used as negative control while DNA from in vitro cultures of *A. phagocytophilum*, *Anaplasma* sp. ‘Omatjenne’, *A. marginale* and *E. ruminantium* were used as positive control. Negative samples were retested at 1/10 dilution to check if a high concentration of DNA inhibited the reaction.

**Table 2:** List of primers used for PCR amplifications and their sequences

Primer	Sequence (5′-3′)	Target gene	Size (bp)	Reference
HER 16SD	GGTACCYACAGAAGAAGTCC	*16S* rDNA	925	[20]
EBR_2_	TGCTGACTTGACATCATCCC			[16]
EBR_3_	TTGTAGTCGCCATTGTAGCAC			[16]
AB128	ACTAGTAGAAATTGCACAATCTAT	*pCS20*	280	[21]
AB129	TGATAACTTGGTGCGGGAAATCCTT			[21]
ITM130	TCAATTGCTTAATGAAGCACTAACTCAC			[21]

Identification to species level of the detected *Anaplasma* was carried out by using restriction with *Mbo*II (5000 U/ml, Biolabs, New England, USA), *Hha*I (20,000 U/ml, Biolabs) and *Msp*I (20,000 U/ml, Biolabs) as described earlier by [9]. Restriction was carried out in a final volume of 15 μl mix consisting of 4 μl DNA (PCR product from positive samples) and 11 μl reaction mix. Incubation was done overnight following the manufacturer’s instructions. The restricted fragments were separated on 2% high resolution agarose gel by electrophoresis in TAE buffer (0.04 M Tris, 0.4 mM EDTA, pH = 7.7–8.8) at 100 V for 40 min and visualised under UV illumination after staining with ethidium bromide (final concentration of 0.5 μg/ml).

#### Identification of *E. ruminantium* using pCS20 PCR

A semi-nested *pCS20* PCR assay was used to identify *E. ruminantium* DNA on randomly selected samples and samples collected from clinical cases of heartwater in Ethiopia. The assay was carried out as described by [25, 26] using AB129 and ITM130 as external reverse and forward primers, and AB128 and AB129 as internal primers (Table 2). After initial denaturation of DNA at 94 °C for 3 min, the first round of amplification was carried out using 40 cycles of 30s denaturation at 94 °C, 45 s annealing at 62 °C, 1 min elongation at 72 °C and a final extension of 10 min at 72 °C. For the second round amplification, 0.5 μl of the PCR product of the first round amplification was used as template. The amplification process consisted of 25 cycles of the same PCR conditions as in the first round except that the annealing temperature was set at 58 °C. The amplification process was carried out in the thermocycler described above. The presence of *E. ruminantium* DNA was analysed using a 2% agarose gel electrophoresis, giving a 280 bp DNA fragment for a positive sample. A PCR mix without DNA template was used as a negative control while DNA from *E. ruminantium* was used as a positive control.

## Results

### *Anaplasma phagocytophilum* and *Anaplasma* sp. ‘Omatjenne’ survey

The results of the international survey and the results of the Ethiopian survey are combined for clarity. A total of 695 samples from cattle were assayed for the presence of DNA of *A. phagocytophilum* and *Anaplasma* sp. ‘Omatjenne’. Of these, 19 (2.7%) and 45 (6.5%) yielded positive signals, respectively (Table 3). *Anaplasma* sp. ‘Omatjenne’ was detected in all countries except Tanzania while *A. phagocytophilum* was detected only in samples originating from Ethiopia.

**Table 3:** Results of molecular analysis of bovine samples from selected African countries for infection with *Anaplasma* sp. ‘Omatjenne’ and *A. phagocytophilum*

Country	No. tested	*A. phagocytophilum*		*Anaplasma* sp. ‘Omatjenne’	
		No. positive	Prevalence (95% CI)	No. positive	Prevalence (95% CI)
Ivory Coast	53	0	0 (0.000–0.055)	2	0.038 (0.004–0.130)
Morocco	80	0	0 (0.000–0.036)	1	0.012 (0.001–0.067)
Rwanda	50	0	0 (0.000–0.058)	8	0.160 (0.072–0.291)
Zambia	37	0	0 (0.000–0.078)	5	0.135 (0.045–0.288)
Tanzania	17	0	0 (0.000–0.162)	0	0 (0.000–0.162)
Ethiopia					
Bako	149	16	0.17 (0.063–0.169)	13	0.087 (0.047–0.144)
Bishoftu	125	3	0.024 (0.005–0.069)	11	0.088 (0.045–0.152)
Habernosa	145	0	0.000 (0.000–0.020)	5	0.034 (0.011–0.079)
Alage	38	0	0.000 (0.000–0.076)	0	0.000 (0.000–0.076)
Overall	457	19	0042 (0.025–0.064)	29	0.063 (0.043–0.090)
Total	695	19	0.027 (0.017–0.042)	45	0.065 (0.048–0.086)

### Oromia survey

#### Identification of *Ehrlichia* spp. and *Anaplasma* spp. using molecular tools

Results of the molecular analysis are given in Table 4. Out of the 922 blood samples from cattle, sheep and goats from five different localities analysed by *16S* rDNA PCR, 523 (56.7%) tested positive. The lowest proportion of positive samples was observed at Habernosa (44%) while the highest was observed at Alage (79%). None of the samples collected from Adami-Tullu yielded a positive result. The variation in the proportion of positive samples among the locations could not be compared statistically as the species composition (and thus the resulting sample) in the five livestock premises was not the same. The highest proportion of positive samples was observed in cattle followed by sheep and the lowest in goats. Overall, 67.4% of cattle, 65% of sheep and 18% of goats tested positive for one or more *Anaplasma* spp. The difference in prevalence among the species could again not be tested statistically, as an effect of sampling location could not be excluded (e.g. a comparison at Bishoftu, the only location where all three species were sampled, was not significant). Mixed infections, mostly with *A. marginale* and *A. centrale* were detected at all four sites with a proportion of 11% at Bishoftu, 13.4% at Bako, 15% at Habernosa and 15.8% at Alage.

**Table 4:** Number and percentage of samples tested positive by *16S* rDNA in cattle, sheep and goats in five sites in central Oromia, Ethiopia (*n* = 922)

	Bako	Alage	A/Tullu	Habernosa	Bishoftu
Cattle					
Number tested	149	38	–	145	125
Number positive	99	30	–	104	75
Proportion (%)	66.4	79	–	71.7	60
Sheep					
Number tested	164	–	–	–	125
Number positive	124	–	–	–	63
Proportion (%)	75.6	–	–	–	48
Goats					
Number tested	–	–	20	105	51
Number positive	–	–	0	5	26
Proportion (%)	–	–	0	4.8	51
Overall (%)	71.3	79	0	43.6	53.5

Restriction fragment length polymorphism (RFLP) analysis resulted in the diagnosis of *A. marginale*, *A. centrale*, *A. phagocytophilum*, *A. ovis* and *Anaplasma* sp. ‘Omatjenne’ (Table 5). This is the first report of the infection of domestic ruminants with *A. phagocytophilum*, *A. ovis* and *Anaplasma* sp. ‘Omatjenne’ in Ethiopia. The occurrence of *A. phagocytophilum* together with *Anaplasma* sp. ‘Omatjenne’ outside Europe and South Africa was observed for the first time. The proportion of samples that tested positive for *A. marginale* was higher at Bako and Alage than at Habernosa and Bishoftu. The occurrence of *A. centrale* was highest at Alage and lowest at Bako. Only samples from Bako and Bishoftu gave positive results for *A. phagocytophilum* and *A. ovis* and the proportion of samples positive for both pathogens was higher at Bako than at Bishoftu. Almost the same numbers of positive samples for *Anaplasma* sp. ‘Omatjenne’ were found at Bako and Bishoftu, and half of these numbers at Habernosa.

**Table 5:** Frequency of infection with *Anaplasma* spp. in cattle, sheep and goats in five livestock premises in central Oromia, Ethiopia

Location	Number (%) positive for				
	*AM*	*AspO*	*APH*	*AC*	*AO*
Bako					
Cattle (*n* = 149)	67 (44.97)	13 (8.72)	16 (10.74)	14 (9.40)	0 (0)
Sheep (*n* = 164)	120 (73.17)	10 (6.09)	2 (1.22)	3 (1.83)	26 (15.85)
Alage					
Cattle (*n* = 38)	23 (60.53)	0 (0)	0 (0)	11 (28.95)	0 (0)
Habernosa					
Cattle (*n* = 145)	67 (46.21)	5 (3.45)	0 (0)	50 (34.48)	0 (0)
Goats (*n* = 105)	3 (2.86)	2 (1.90)	0 (0)	0 (0)	0 (0)
Bishoftu					
Cattle (*n* = 125)	45 (36.00)	11(8.80)	3 (2.40)	48 (38.40)	0 (0)
Sheep (*n* = 125)	27 (21.60)	5 (4.00)	4 (3.20)	7 (5.60)	22 (17.60)
Goats (*n* = 51)	15 (29.41)	1 (1.96)	3 (5.88)	5 (9.80)	4 (7.84)
A/Tullu					
Goats (*n* = 20)	0 (0)	0 (0)	0 (0)	0 (0)	0 (0)
Overall (*n* = 922)	367 (39.80)	47 (5.10)	28 (3.00)	138 (15.00)	52 (5.60)

*Abbreviations*: *AM* *A. marginale*, *AspO* *Anaplasma* sp. ‘Omatjenne’, *APH* *A. phagocytophilum*, *AC* *A. centrale*, *AO* *A. ovis*

#### Results of pCS20 PCR amplification

Since none of the *16S* rDNA PCR-RFLP analyses yielded positive results for *E. ruminantium*, *pCS20* PCR was used to analyse randomly selected samples. Thus, from the 922 DNA samples, 493 samples (271 cattle, 145 sheep and 77 goats) were randomly selected and tested for *E. ruminantium*. Three of the 75 cattle blood samples tested from Bako gave a positive result giving an apparent prevalence of 4% in the area. Overall, the prevalence of *E. ruminantium* in the cattle included in this study was 0.6%.

#### Active clinical search

The prevalence of *E. ruminantium* detected was very low. This is contrary to the fact that several outbreaks of heartwater have been reported based on clinical observations. This active clinical search was done to check if the outbreaks are caused by *E. ruminantium* or other pathogens. Thirteen blood samples (ten from the dairy farm in Bishoftu and three from the dairy farm of Haramaya University) were collected from clinically affected animals and analysed by *pCS20* PCR. Three (30%, 95% CI: 6.7–65.2%) and two (66.7%, 95% CI: 9.4–99.2%) of the samples from Bishoftu and Haramaya University, respectively, tested positive for *E. ruminantium*. Overall 38.46% (95% CI: 13.86–68.42) of the clinically affected cows were found positive.

## Discussion

Since it was first recognised, anaplasmosis caused by *A. phagocytophilum* is considered to have a worldwide distribution. However, studies about the extent of its occurrence in animals and humans have been limited to Europe and the USA. Advances in molecular diagnosis have resulted in new evidence for the wide occurrence of this pathogen in the Northern Hemisphere [27]. In Africa, where many tick-borne diseases are encountered [28], data that support the occurrence of *A. phagocytophilum* is lacking, possibly due to lack of appropriate diagnostic tools: diagnosis of anaplasmosis, based on microscopic examination of stained blood smears and serologic tests, is unable to differentiate the various species and requires a high level expertise and skill [3, 29]. In this study we used molecular methods to investigate the occurrence of *A. phagocytophilum* in Africa.

So far no reports of clinical cases associated with *A. phagocytophilum* have been published in Africa. Very few studies have reported its occurrence in animals and ticks within the continent. Overall, the proportion of infected animals reported by earlier authors is consistent with our findings. For example, the overall prevalence observed in this study (2.73%) agrees with the prevalence reported by Muhanguzi et al. [7] in cattle (2.70%) from Uganda and Teshale et al. (unpublished data) in sheep and goats (2.49%) from Ethiopia. The present result is higher than the prevalence reported by M’Ghirbi et al. [30] in horses (0.90%) in Tunisia, where the same authors do, however, report a prevalence of 13% in dogs [31]. The apparent variability in the prevalence of *A. phagocytophilum* can, in the first instance, be explained by the different host species examined and differences in environmental conditions between Tunisia and Ethiopia also cannot be excluded; Tunisia is characterised by a mediterranean/subtropical climate while Ethiopia features tropical conditions, a difference that affects tick species, their numbers, distribution and infection rate with tick-borne pathogens [32]. *Anaplasma phagocytophilum* has also been detected in ticks in Africa: in questing *Rhipicephalus pulchelus* in Ethiopia [33] and in *I. ricinus* in Tunisia [8]. It must be noted that higher prevalence levels of *A. phagocytophilum* have been reported outside Africa, e.g. 16.7% cattle in Italy [34]. Furthermore, the prevalence, based on the detection of DNA, was shown to be as high as 51% in Guatemala, 20% in France, 13–19% in Spain and 1–80% in Japan [3].

In the present study, no samples collected from Ivory Coast, Morocco, Rwanda, Tanzania and Zambia were positive for *A. phagocytophilum*, possibly due to the small sample sizes tested, collected only from cattle (95% confidence intervals shown in Table 3; all include expected prevalence levels). Secondly, it could be due to the low level of *A. phagocytophilum* circulating in the persistently infected hosts, characterised by intermittent periods of bacteraemia; earlier studies have shown that domestic animals are persistently infected with *A. phagocytophilum* causing cyclic bacteraemia featured by periodic peaks [35]. Hence, sampling done during the periods when there is no or low bacteraemia would yield negative results. The effect of the distribution of vectors and reservoirs on the occurrence of *A. phagocytophilum* in domestic animals was discussed recently [36]. Absence of positive samples in the above African countries does not, therefore, rule out the occurrence of this pathogen. Further work involving larger samples is needed to clarify this situation.

Infection with *Anaplasma* sp. ‘Omatjenne’ was recognised for the first time in cattle in an *Amblyomma*-free farm in Namibia. At that time it was shown that 81% of the cattle on the farm were seropositive to heartwater but no evidence of clinical cases of the disease was found. The extent of its occurrence and importance was then not explored, mainly due to a lack of differential diagnosis [37], a situation that has changed since PCR-RFLP targeting *16S* rDNA was able to differentiate between this pathogen and other *Anaplasma* spp. [9]. Infection with this pathogen was detected in samples collected from Ivory Coast, Morocco, Rwanda, Zambia and Ethiopia but not from Tanzania. The prevalence of samples that tested positive ranged from 1.23% in Morocco to 16% in Rwanda with an overall average of 6.47%. This shows that it is widespread in African countries. The proportion of samples from Morocco giving a positive signal is similar to reports of Muhanguzi et al. [7] and Aktas et al. [12]. In contrast, the proportion of samples from Ivory Coast, Rwanda, Zambia and Ethiopia testing positive is higher, agreeing with previous reports [14]. This variation may be due to differences in sampling strategy, sampling season and cattle breeds sampled. The difference in the sensitivity of the assay methods used in this study, compared to previous work [7, 14], could also be another factor that contributes to the variation.

In this study we provide the first molecular evidence for the occurrence of infections with *E. ruminantium* and five *Anaplasma* spp. (*A. marginale*, *A. phaocytophilum*, *A. ovis*, *A. centrale* and *Anaplasma* sp. ‘Omatjenne’) in domestic ruminants in Ethiopia. To our knowledge this is the first report of the presence of *A. phagocytophilum*, *A. ovis* and *Anaplasma* sp. ‘Omatjenne’ infection in domestic ruminants in the country. *Anaplasma* spp. were more prevalent than *Ehrlichia* spp. at all sites and in all animal species. Overall more than half of the animals examined tested positive for *Anaplasma* spp. and the proportion might even be higher during the wet season. Even though samples were collected from apparently healthy animals, infections with these pathogens are not without negative effects. *Anaplasma* spp. have been known to cause a reduction in body weight, milk yield, abortions in pregnant animals [5] and immune-suppression in infected animals [38]. Moreover, it has been shown that the outcome of infection with *Anaplasma* spp. can be more severe in the presence of co-infection [39]. In line with this we detected mixed infections at all sites during this study.

Results of earlier serologic surveys for anaplasmosis in cattle in Ethiopia showed a prevalence ranging from 84% [18] to 99% [22], which is higher than our observation. This may be attributed to the persistence of antibody at detectable levels among infected and immune animals for longer periods of time than rickettsiaemia. Differences in sampling season and management of animals might be other causes of variation in the prevalence of infection. The previous studies were carried out during the rainy season whilst our study was done during the dry season. However, our study reports a much higher prevalence of anaplasmosis than the 2.2% reported using microscopy [22]. The overall prevalence of infection with *Anaplasma* spp. observed in this study is higher than previous reports from Kenya [40, 41], Tanzania [42], Uganda [7] and Turkey [12], probably due to a difference in environmental factors and management practices. Our finding is comparable to results of earlier authors from Zambia [43] and Costa Rica [44]. The prevalence of infection with *A. marginale* reported in this study is also comparable to the earlier reports in Italy [34] and Ghana [45]. The prevalence of *A. marginale* reported in this study is, however, higher than the earlier reports [12, 46]. The prevalence of *A. ovis* reported in this study is lower that the prevalence reported previously [34, 47–49]. In Ethiopia, the agro-climatic conditions are favorable for many tick species and no tick or tick-borne disease control is practiced. Previous field studies showed that more than 60 species of ticks are present in the country [50]. The dominant production system is extensive resulting in free contact between livestock and wildlife. The absence of tick control allows infestation of domestic animals by ticks and free ranging domestic animals that share grazing areas with wildlife would increase the chance of circulation of tick-borne pathogens. Kocan et al. [51] (but also [4, 52]) pointed out that the ultimate reservoir of tick-borne rickettsia is wildlife. Within Ethiopia, higher prevalence of *Anaplasma* spp. were recorded in wetter, more humid localities, favouring higher densities of ticks (e.g. Bako), and localities where exotic animals are kept (e.g. Alage). This is consistent with earlier reports [43, 51]. The higher prevalence of *A. ovis* at Bako and Bishoftu is most likely due to the sampling of sheep in these areas whereas no sheep were sampled at the other sites.

*Anaplasma* sp. ‘Omatjenne’ was detected at three localities within Ethiopia. It was also detected in five out of six African countries. Although it has been suggested to be apathogenic to cattle by some authors [11, 52], the parasite seems to be widespread. Its occurrence in cattle on farms and ranches where outbreaks of tick-borne diseases were reported on the basis of clinical and postmortem examination warrant further epidemiological studies. The proportion reported for *Anaplasma* sp. ‘Omatjenne’ in this study is higher than that of [7, 12]. As explained before, such variability in the proportions could arise from differences in season of study, management of study animals and type and frequency of control activities. The previous detection of infection with this pathogen in the African buffalo (*Syncerus caffer*) [14] and in the nyala (*Tragelaphus angasii*) [13] confirms the occurrence of natural infection in these wildlife species.

Systematic investigations of heartwater are not undertaken in Ethiopia. The occurrence of the disease has been suspected either on the basis of clinical signs or based on brain squash smear examination. We provided the first molecular evidence of infection with *E. ruminantium* in blood samples collected from ruminants. It was detected in 0.6% of samples from cattle. Previously, DNA belonging to this pathogen was detected in *Amblyomma* spp. with a prevalence ranging from 0.9 to 11.67% [9]. The proportion of samples that tested positive for *E. ruminantium* in this study is lower than the previous reports [7, 26, 43, 53–55]. Although ruminants remain the primary mammalian hosts of *E. ruminantium*, possible canine infection [56] and its association with several cases of rapidly fatal encephalitis in humans [52] in South Africa, raises the question about the importance of this pathogen in causing disease in pets and humans. Hence, this study provides preliminary information for the veterinary and public health authorities for further investigation. *Anaplasma phagocytophilum* has been shown to cause clinical disease and impair ruminant production [38] and human granulocytic anaplasmosis [4]. The occurrence of *A. phagocytophilum* in domestic animals and ticks raises the question of whether human granulocytic anaplasmosis occurs in Africa in general and in Ethiopia in particular. The absence of published clinical cases caused by *A. phagocytophilum* in the continent, however, could be due to lack of differential diagnosis of febrile diseases other than malaria.

## Conclusions

The occurrence of both *Anaplasma* sp. ‘Omatjenne’ and *A. phagocytophilum* in African cattle is confirmed. Although humans may be bitten by ticks or biting flies carrying zoonotic anaplasmas, the public health implication of *A. phagocytophilum* in Africa remains to be elucidated. Similarly the occurrence of *Anaplasma* sp. ‘Omatjenne’ infection in cattle in Africa raises the question of whether or not it is involved in clinical diseases. Livestock improvement plans such as smallholder dairy development schemes through introduction of high yielding breeds should be aware of the importance of these diseases and take the necessary precautions to avoid losses. Further epidemiological investigation of tick-borne diseases is needed to fully understand their impact. Detailed studies on *Anaplasma* sp. ‘Omatjenne’ infections and the public health roles of *A. phagocytophilum* and *A. ovis* infection are recommended.

## References

[1] Dulmer JS, Barbet AF, CPJ B, Dasch GA, Palmer GH, Ray SC (2001). Reorganisation of genera in the families of Rickettsciaceae and Anaplasmataceae in the order Rickettsiales: unification of some species of *Ehrilichia* with *Anaplasma*, *Cowdria* with *Ehrlichia* and *Ehrlichia* with *Neorickettscia*, descriptions of six new species combinations and designation of *Ehrlichia equi* and `HGE agent' as subjective synonyms of *Ehrlichia phagocytophilia*. Int J Syst Evol Biol.

[2] Yoo-eam S (2012). Molecular epidemiology of tick-borne Anaplasmataceae among client-owned dogs in Missouri.

[3] Stuen S, Granquist EG, Silaghi C (2013). *Anaplasma phagocytophilum* - a widespread multi-host pathogen with highly adaptive strategies. Frontiers Cell Infect Microbiol.

[4] Woldehiwet Z (2010). The natural history of *Anaplasma phagocytophilum*. Vet Parasitol.

[5] Rymaszewska A, Grenda S (2008). Bacteria Of the genus *Anaplasma* - characteristics of *Anaplasma* and their vectors: a review. Veterinarni Medicina.

[6] Santos AS, Santos-Silva MM, Almeida VC, Bacellar F, Dumler JS (2004). Detection of *Anaplasma phagocytophilum* DNA in Ixodes ticks (Acari: Ixodidae) from Madeira Island and Setubal District, mainland Portugal. Emerg Infect Dis.

[7] Muhanguzi D, Ikwap K, Picozzi K, Waiswa C. Molecular characterization of *Anaplasma* and *Ehrlichia* species in different cattle breeds and age groups in Mbarara District (western Uganda). Int J Anim vet Advances. 2010;2:76–88.

[8] Sarih M, M'Ghirbi Y, Bouattour A, Gern L, Baranton G, Postic D (2005). Detection and identification of *Ehrlichia* spp. in ticks collected in Tunisia and Morocco. J Clin Microbiol.

[9] Teshale S, Geysen D, Ameni G, Asfaw Y, Berkvens D. Improved molecular detection of *Ehrlichia* and *Anaplasma* species applied to *Amblyomma* ticks collected from cattle and sheep in Ethiopia. Ticks Tick-borne Dis. 2015;6:1–7. 10.1016/j.ttbdis.2014.04.023 25438799

[10] Anderson AD, Smoak B, Shuping E, Ockenhouse C, Petruccelli B (2005). *Anaplasma phagocytophilum* in Sardinia, Italy. Emerg Infect Dis.

[11] Allsopp BA, Allsopp MT, Visser ES, Du Plessis JL, Vogel SW (1997). Different organisms associated with heartwater as shown by analysis of *16S* ribosomal RNA gene sequences. Vet Parasitol.

[12] Aktas M, Altay K, Dumanli N (2011). Molecular detection and identification of *Anaplasma* and *Ehrlichia* species in cattle from Turkey. Ticks Tick-borne Dis.

[13] Pfitzer S, Ooshuizen MC, Bosman AM, Voster I, Penzhorn BL. Tick-borne blood parasites in nyala (*Tragelaphus * *angasii*, Gray 1849) from Kuwa Zulu-Natal, South Africa. Vet Parasitol. 2011;176:126–31. 10.1016/j.vetpar.2010.11.006 21145660

[14] Debeila EM (2012). Occurrence of *Anaplasma* and *Ehrlichia* species in African buffaloes (*Syncerus caffer*) in Kruger National Park and Hluhluwe-iMfolozi park in South Africa.

[15] Pegram R, Hoogstraal H, Wassef H (1981). Ticks (Acari: Ixodoidea) of Ethiopia. Distribution, ecology and host relationship of species infesting livestock. Bull Entomol Res.

[16] Zeleke B, Bekele T (2010). Species of ticks on camel and their seasonal dynamics in eastern Ethiopia. Trop Anim Hlth Prod..

[17] Abera M, Mohammed T, Abebe R, Aragaw K, Bekele J (2010). Survey of ixodid ticks in domestic ruminants in Bedelle district, southwestern Ethiopia. Trop Anim Hlth Prod.

[18] Feleke A, Petros B, Lemecha H, Wossene A, Mulatu W, Rege EJO. Study on monthly dynamics of ticks and seroprevalence of *Anaplasma marginale*, *Babesia bigemina* and *Theileria mutans* in four indigenous breeds of cattle in Ghibe Valley, Ethiopia. SINET: Ethiopian J Sci. 2008;31:11–20.

[19] Bose R, Jorgensen W, Dalgliesh R, Friedhoff K, de Vos A (1995). Current state and future trends in the diagnosis of babesiosis. Vet Parasitol.

[20] Tomassone L, Grego E, Call_a G, Rodighiero P, Pressi G, Gebre S (2012). Ticks and tick-borne pathogens in livestock from nomadic herds in the Somali region, Ethiopia. Exp Appl Acarolo.

[21] Kumsa B, Signorini M, Teshale S, Tessarin C, Duguma R, Ayana D (2013). Molecular detection of piroplasms in ixodid ticks infesting cattle and sheep in western Oromia, Ethiopia. Trop Anim Hlth Prod..

[22] Mekonnen S. Epidemiology of ticks and tick-borne diseases in Ethiopia: future research needs and priorities. In: Irvin AD, McDermott JJ, Perry BD, editors. Epidemiology of ticks and tick-borne diseases in eastern, central and southern Africa. Nairobi: International Livestock Research Institute; 1996.

[23] Walker AR, Bouattour A, L CJ, Estrada-Pena A, Horak IG, Pegram RG, et al. Ticks of domestic animals in Africa: a guide to identification of species. Edinburgh: Bioscience Reports; 2003.

[24] Hornok S, Foldvari G, Elek V, Naranjo V, Farkas R, Fuente J (2008). Molecular identification of *Anaplasma marginale* and rickettsial endosymbionts in blood-sucking flies (Diptera: Tabanidae, Muscidae) and hard ticks (Acari: Ixodidae). Vet Parasitol.

[25] Martinez D, Vachiery N, Stachurski F, Kandassamy Y, Raliniaina M, et al. Nested PCR for detection and genotyping of *Ehrlichia ruminantium*: use in genetic diversity analysis. Ann NY Acad Sci. 2004;1026:106–13. 10.1196/annals.1307.014 15604477

[26] Faburay B, Geysen D, Munstermann S, Bell-Sakyi L, Jongejan F. Longitudinal monitoring of *Ehrlichia ruminantium* infection in Gambian lambs and kids by *pCS20* PCR and MAP1-B ELISA. BMC Infect Dis. 2007;7(85). 10.1186/1471-2334-7-85 PMC1949406 17662144

[27] Rymaszewska A (2011). PCR For detection of tick-borne *Anaplasma phagocytophilum* pathogens: a review. Veterinarni Medicina..

[28] Kocan KM, Blouin EF, Barbet AF (2000). Anaplasmosis control. Past, present, and future. Ann NY Acad Sci.

[29] Simuunza MC. Differential diagnosis of tick-borne diseases and population genetic analysis of *Babesia bovis* and *Babesia bigemina*. Pretoria University; 2009.

[30] M'Ghirbi Y, Yaich H, Ghorbel A, Bouattour A (2012). *Anaplasma phagocytophilum* in horses and ticks in Tunisia. Parasit Vectors.

[31] M'Ghirbi Y, Ghorbel A, Amouri M, Nebaoui A, Haddad S, Bouattour A (2009). Clinical, serological, and molecular evidence of ehrlichiosis and anaplasmosis in dogs in Tunisia. Parasitol Res.

[32] de la Fuente J, Estrada-Peña A, Venzal JM, Kocan KM, Sonenshine DE. Overview: ticks as vectors of pathogens that cause disease in humans and animals. Front Biosci. 2008;(13):6938–46. 10.2741/3200 18508706

[33] Teshale S, Geysen D, Ameni G, Ketema B, Dorny P, Berkvens D (2016). Molecular detection of *Anaplasma* species in questing ticks (ixodids) in Ethiopia. Asian Pacific J Trop Dis.

[34] Torina A, Vicente J, Alongi A, Scimeca S, Turlá R, Nicosia S (2007). Observed prevalence of tick-borne pathogens in domestic animals in Sicily, Italy during 2003–2005. Zoonoses Pub Hlth.

[35] Ladbury GAF, Stuen S, Thomas R, Bown KJ, Woldehiwet Z, Granquist EG (2008). Dynamic transmission of numerous *Anaplasma phagocytophilum* genotypes among lambs in an infected sheep flock in an area of anaplasmosis endemicity. J Clin Microbiol.

[36] Baráková I, Derdáková M, Carpi G, Rosso F, Collini M, Tagliapietra V (2014). Genetic and ecologic variability among *Anaplasma phagocytophilum* strains, northern Italy. Emerg Infect Dis.

[37] Holland CJ, Logan LL, Mebus EA, Ristic M (1987). The serological relationship between *Cowdria ruminantium* and certain members of the genus *Ehrlichia*. Onderstepoort J Vet Res.

[38] Larsen HJS, Overas G, Waldeland H, Johansen GM (1994). Immunosuppression in sheep experimentally infected with *Ehrlichia phagocytophila*. Res Vet Sci.

[39] Renneker S, Abdo J, Salih DEA, Karagen_c T, Bilgi_c H, Torina A (2013). Can *Anaplasma ovis* in small ruminants be neglected any longer?. Transboundary Emerg Dis.

[40] Okuthe O, Buyu G (2006). Prevalence and incidence of tick-borne diseases in smallholder farming systems in the western-Kenya highlands. Vet Parasitol.

[41] Muraguri G, McLeod A, McDermott J, Taylor N (2008). The incidence of calf morbidity and mortality due to vector-borne infections in smallholder dairy farms in Kwale District, Kenya. Vet Parasitol.

[42] Swai ES, Karimuribo ED, Kambarage DM, Moshy WE (2009). A longitudinal study on morbidity and mortality in young stock smallholder dairy cattle with special reference to tick-borne infections in Tanga region, Tanzania. Vet Parasitol.

[43] Simuunza M, Weir W, Courcier E, Tait A, Shiels B (2011). Epidemiological analysis of tick-borne diseases in Zambia. Vet Parasitol.

[44] Oliveira JB, Montoya J, Romero JJ, Urbina A, Soto-Barrientos N, Melo ESP (2011). Epidemiology of bovine anaplasmosis in dairy herds from Costa Rica. Vet Parasitol.

[45] Bell-Sakyi L, Koney EBM, Dogbey O, Walker AR. *Ehrlichia ruminantium* seroprevalence in domestic ruminants in Ghana; I. Longitudinal survey in the Greater Accra region. Vet Microbiol 2004;100:175–188. 10.1016/j.vetmic.2004.02.010 15145496

[46] Awad H, Antunes S, Galindo RC, do Ros_ario VE, De la Fuente J, Domingos A (2011). Prevalence and genetic diversity of *Babesia* and *Anaplasma* species in cattle in Sudan. Vet Parasitol.

[47] Hornok S, Elek V, De la Fuente J, Naranjo V, Farkas R, Majoros G (2007). First serological and molecular evidence on the endemicity of *Anaplasma ovis* and *A. marginale* in Hungary. Vet Microbiol.

[48] Razmi GR, Dastjerdi K, Hosseini H, Naghibi A, Barati F, Aslani MR. An epidemiological study on *Anaplasma* infection in cattle, sheep and goats in Mashhad suburb, Khorasan Province, Iran. Ann NY Acad Sci. 2006;1078:479–81. 10.1196/annals.1374.089 17114758

[49] Torina A, Galindo RC, Vicente J, Marco V, Russo M, Aronica V (2010). Characterization of *Anaplasma phagocytophilum* and *A. ovis* infection in a naturally infected sheep flock with poor health condition. Trop Anim Hlth Prod..

[50] Morel PC (1980). Study on Ethiopian ticks (acarida, Ixodidae).

[51] Kocan KM, de la Fuente J, Blouin EF, Coetzee JF, Ewing SA (2010). The natural history of *Anaplasma marginale*. Vet Parasitol.

[52] Allsopp BA (2010). Natural history of *Ehrlichia ruminantium*. Vet Parasitol.

[53] Awad D. Serological survey of heartwater relative to the distribution of the vector *Amblyomma variegatum* and other tick species in North Cameroon. Vet Parasitol. 1997;68:165–73. 10.1016/s0304-4017(96)01058-8 9066062

[54] Bekker CP, Vink D, Lopes Pereira CM, Wapenaar W, Langa A, Jongejan F (2001). Heartwater (*Cowdria ruminantium* infection) as a cause of post restocking mortality of goats in Mozambique. Clin Diagn Lab Immunol.

[55] Koney EBM, Dogbey O, Walker AR, Bell-Sakyi L (2004). *Ehrlichia ruminantium* seroprevalence in domestic ruminants in Ghana. II. Point prevalence survey. Vet Microbiol.

[56] Allsopp MT, Allsopp BA (2001). Novel *Ehrlichia* genotype detected in dogs in South Africa. J Clin Microbiol.

